# Green cardamom increases Sirtuin-1 and reduces inflammation in overweight or obese patients with non-alcoholic fatty liver disease: a double-blind randomized placebo-controlled clinical trial

**DOI:** 10.1186/s12986-018-0297-4

**Published:** 2018-09-25

**Authors:** Milad Daneshi-Maskooni, Seyed Ali Keshavarz, Mostafa Qorbani, Siavash Mansouri, Seyed Moayed Alavian, Mahtab Badri-Fariman, Seyed Ali Jazayeri-Tehrani, Gity Sotoudeh

**Affiliations:** 1 Department of Community Nutrition, School of Nutritional Sciences and Dietetics, Tehran University of Medical Sciences, No.44, Hojjatdoust Alley, Naderi Ave, Keshavarz Blvd, Tehran, 1416643931 Iran; 2 Department of Clinical Nutrition, School of Nutritional Sciences and Dietetics, Tehran University of Medical Sciences, Tehran, Iran; 3 Non-Communicable Diseases Research Center, Alborz University of Medical Sciences, Karaj, Iran; 4 Gastroenterohepatology Department, National Iranian Oil Company (NIOC) Central Hospital, Tehran, Iran; 5 Baqiyatallah Research Center for Gastroenterology and Liver Diseases (BRCGL), Baqiyatallah University of Medical Sciences, Tehran, Iran

**Keywords:** Non-alcoholic fatty liver disease, Green cardamom, Overweight or obesity, Sirtuin-1, Inflammatory factors

## Abstract

**Background:**

Non-alcoholic fatty liver disease (NAFLD) is the hepatic component of metabolic syndrome. Despite the beneficial health effects of cardamom on dyslipidemia, hepatomegaly, and fasting hyperglycemia, no previous human study has been conducted on the efficacy of cardamom in NAFLD. The aim of this study was to assess the effects of green cardamom (GC) on serum Sirtuin-1 (Sirt1), inflammatory factors, and liver enzymes in overweight or obese NAFLD patients.

**Methods:**

The recruitment of subjects was conducted at the polyclinic of the central hospital of National Iranian Oil Company (NIOC), Tehran. Eighty-seven patients who participated were divided randomly into two groups according to the ultrasonography and eligibility criteria as cardamom (*n* = 43) or placebo (*n* = 44). The intervention involves taking two 500 mg capsules three times per day with meals for 3 months. General characteristics, dietary intake and physical activity status, weight and height were determined. In addition, serum Sirt1, tumor necrosis factor-alpha (TNF-α), high sensitive c-reactive protein (hs-CRP), interleukin-6 (IL-6), alanine transaminase (ALT), and aspartate transaminase (AST) were measured. The degree of fatty liver was determined at beginning and end of the study.

**Results:**

In comparison with placebo, GC significantly increased Sirt1 and decreased hs-CRP, TNF-α, IL-6, ALT, and the degree of fatty liver (*P* < 0.05). The differences in weight, BMI, and AST were not significant (*P* > 0.05).

**Conclusion:**

GC supplementation could improve some biomarkers related to fatty liver including inflammation, ALT, and Sirt1 in overweight/obese NAFLD patients. Further trials on cardamom’s potential are suggested.

**Trial registration:**

Iranian Registry of Clinical Trials, IRCT2015121317254N4. Registered 27/12/2015.

## Background

Non-alcoholic fatty liver disease (NAFLD), as a triglyceride accumulation of more than 5% in hepatocytes [1], is emerging presently [2]. The prevalence is 25.2% globally [3], 65–85% in obese and 15–20% in non-obese adults [4, 5], and between 20 and 40% in Iranian adults [6, 7]. NAFLD ranges from simple hepatic steatosis to non-alcoholic steatohepatitis (NASH) and sometimes cirrhosis. Obesity, impaired blood glucose, hypertension, and hyperlipidemia as characteristics of metabolic syndrome [8, 9], family history, age, severe weight loss, malnutrition, certain medicines or diseases [10], and enteric microbiota [11] are some risk factors of NAFLD. In simple words, NAFLD is the hepatic component of metabolic syndrome [12]. The inflammatory cytokines, oxidative stress and subsequently insulin resistance may play a role in the pathology of this disease [13, 14]. There is a direct correlation between insulin resistance and liver fat content. The activators of nuclear factor κB (NF-κB) including TNF-α, increase the inflammatory cytokines that can subsequently impair insulin sensitivity [4].

The silent information regulators proteins (sirtuins) have either mono-ADP-ribosyltransferase or deacylase activity, including deacetylase, desuccinylase, demalonylase, demyristoylase and depalmitoylase activity. Of the seven identified sirtuins –Sirt1 to Sirt7– in mammals, Sirt1 has been mostly studied. The activation of Sirt1 has different health benefits [15, 16]. Sirt1 as a histone deacetylase-III in humans [17], decreases oxidative stress by activating antioxidant enzymes including superoxide dismutase (SOD) and catalase [18]. It plays important roles in insulin secretion, lipid/glucose/energy metabolism, insulin resistance, inflammatory process, cardiovascular, kidney, and NAFLD diseases [19], mitochondrial and physiological function, and weight reduction [15, 16]. Also, Sirt1 can increase PPAR-γ coactivator-1 alpha (PGC-1α) that suppresses NF-κB [15, 17, 20]. According to some evidence, Sirt1 is downregulated in the liver of NAFLD patients [21].

The dietary polyphenols play important roles as anti-oxidant and anti-inflammatory compounds [22]. GC as a spice contains multiple polyphenols including flavone (luteolin), flavonols (quercetin and kaempferol), and anthocyanidin (pelargonidin) [23] that are NF-κB suppressors [23–26]. The flavonoids and isoflavones (quercetin, resveratrol, and kaempferol) activate PGC-1α [27, 28] and accordingly, GC may affect insulin sensitivity and hepatic steatosis by suppressing oxidative stress and inflammation [29].

Lifestyle changes such as being physically active and losing weight gradually are the common treatment of NAFLD [30, 31]. Because of the poorly characterized pathogenesis of NAFLD and the controversial treatment of it, new therapeutic plans may be effective for the prevention and treatment of NAFLD [32]. The long-term keeping of weight loss is a challenge [33], and so, the new approaches such as change of dietary components may be helpful [34, 35–37]. GC has some beneficial health effects including antihypertensive, antioxidant, fibrinolysis enhancement, gastroprotective, antispasmodic, antibacterial [38], anti-inflammatory, anti-food toxins, anticarcinogenic, carminative, heart improvement, expectorant, diuretic [39], and antiplatelet aggregation [40]. The 1,8-cineole and alpha-terpinyl acetate are the most common agents of cardamom volatile oil. The reported effects for 1,8-cineole as the most extensively studied agent include the increase of apoptosis, the suppressing of prostaglandins, cytokines, nitric oxide, leukotrienes, interleukin-1 beta (IL-1β), TNF-α, inducible-nitric oxide synthase (iNOS), and cyclooxygenase-2 (COX-2), decrease of liver necrosis, vessels relaxation, the muscarinic receptors blocking, and anticholinergic effect [41].

It was supposed that anti-inflammatory, antioxidant, hypolipidemic, and antibacterial effects of GC may improve NAFLD. Due to important and beneficial roles of Sirt1 in different metabolic pathways involved in the metabolic disorders especially fatty liver, many studies have assessed the activation of Sirt1 for effectively preventing the progress of the fatty liver disease [42, 43]. The stimulating Sirt1 secretion in overweight or obese NAFLD patients by GC needs to be investigated. The levels of serum Sirt1 and the GC efficacy in overweight or obese NAFLD patients have not been previously studied. This trial was designed to assess the effect of GC on serum levels of Sirt1, inflammatory factors, and liver enzymes in overweight or obese NAFLD patients.

## Methods

### Study design and subjects

This study was designed as a double-blind randomized placebo-controlled clinical trial, approved by the ethics committee of Tehran University of Medical Sciences as IR.TUMS.REC.1394.791, and registered as IRCT2015121317254N4 on 27/12/2015. The participants were overweight or obese NAFLD patients referring to the sonography section of NIOC central hospital of Tehran. Our study lasted from 8 May 2016 until 17 September 2017.

*Inclusion criteria* were having NAFLD by ultrasonography, 30–60 years old and 25 ≤ BMI < 35 kg/m^2^. *Exclusion criteria* were history of alcohol usage during the past 12 months, inability to cooperate, conditions influencing the liver, secondary NAFLD, disability, uncontrolled hypertension (> 140/90 mmHg), pregnancy or lactation, professional athlete, intake of ursodeoxycholic acid, antihypertensive, statins, probiotics, drugs interacting with cardamom, and antioxidant and multivitamin-mineral supplements during the past 3 months, weight loss for the past 3 months, and taking less than 90% of the study’s supplements [35–37].

### Randomization and intervention

**Fig. 1:**
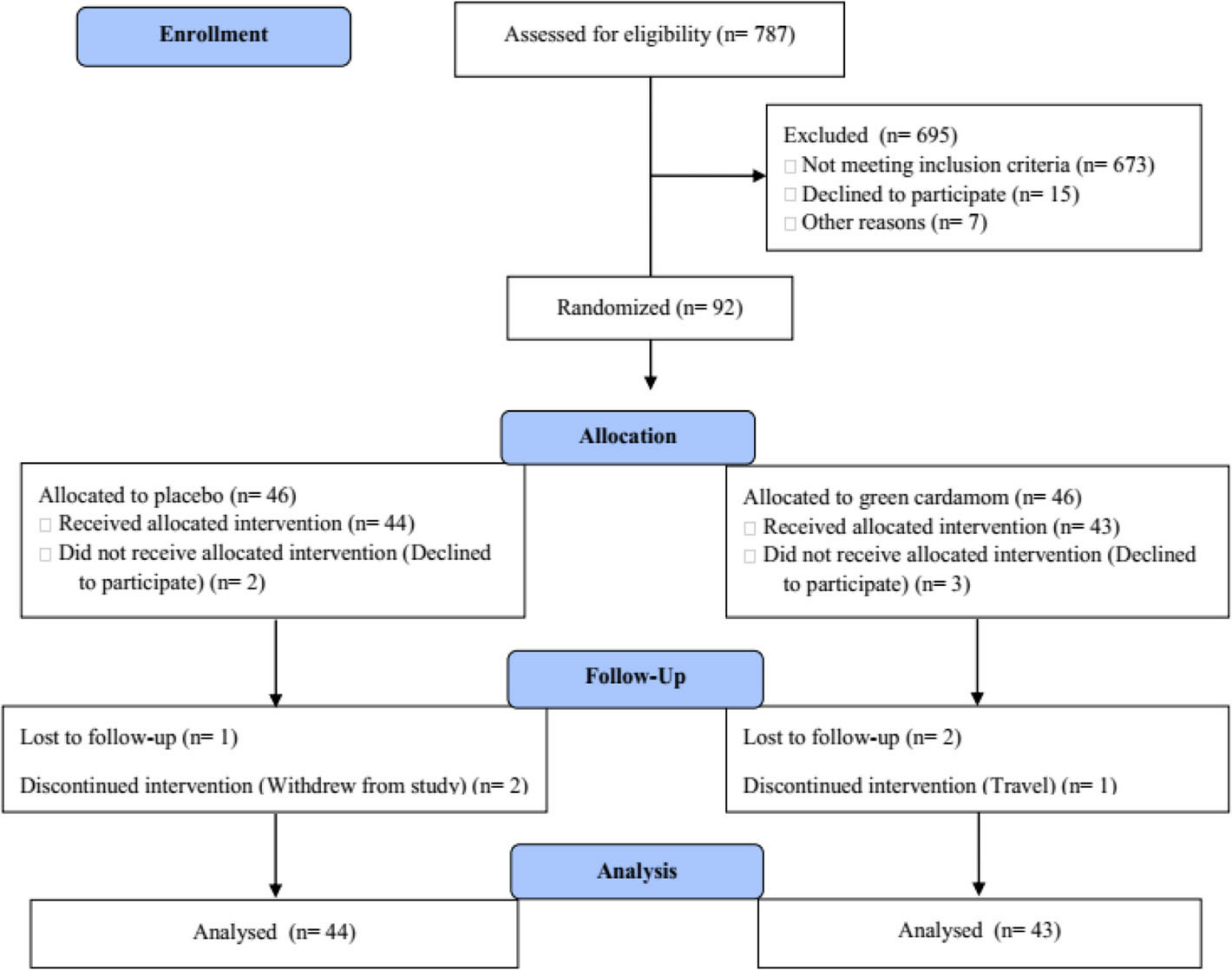
Flow diagram of the study participants

The block randomization method was used to divide patients into two groups, by an assistant (cardamom [*n* = 43] or placebo [*n* = 44]). The stratified randomization was used for controlling age (30–45 and 46–60 yrs) and gender. The ratio of groups was 1:1. After randomization and before the commencement of the study, 3 subjects from GC group and 2 subjects from placebo group declined to participate (Fig. 1).

Both the subjects and investigators were blinded to the intervention allocation. GC was supplied by Samex agency, India. The GC and placebo capsules were similar in shape, size, and color and were prepared by the *Traditional Medicine Research Center (TMRC)***,** Iran University of Medical Sciences, Tehran, Iran. The capsules contained 0.5 g of whole GC or toast flour. Before the intervention, the placebo capsules were placed near the cardamom capsules weekly to have their smell. This absorbed amount of cardamom volatile oil by the placebo capsules is very negligible to affect health parameters. The blinding of supplements for patients and investigators was done through packaging as A and B packs by TMRC. The dose of GC supplement was selected as 3 g/day [38, 44] i.e. 2 capsules with each meal. Similarly, dose of the toast flour as placebo supplement was 3 g/day. The distribution of supplements was once a month and processes such as consumed capsules, potential complications, and the returned packages were checked monthly and by telephone weekly. The recommendations for lifestyle changes were given by an expert dietitian (MDM) placed in the hospital. The process of conducting the trial was checked by an assistant frequently and independently.

The characteristic of GC was *Elettaria cardamomum (L.) Maton, Family: Zingiberaceae, PMP-669*. The whole GC was analyzed by The Institute of Medicinal Plants, Shahid Beheshti University of Medical Sciences, Tehran. The essential oil contents of GC by the gas chromatography-mass spectrometry (GC-MS) were 41% α-terpinyl acetate and 30% 1,8-cineole. The content of total phenolic acid by high-performance liquid chromatography (HPLC) based on standard gallic acid was 10.53 ± 0.18 μg gallic acid equivalent/mg dry extract. Also, the content of total flavonoid by maceration method based on standard quercetin was 4.143 ± 1.865 μg quercetin/mg dry extract.

### Assessments and measurements

#### General characteristics, dietary intakes, and physical activity

After identifying NAFLD patients, the eligibility criteria were checked, the details and benefits of the study were clarified, and informed consent was obtained by the principal investigator. The general questionnaire, 24-h food recall (at the beginning, middle, and end), and short-form IPAQ (SF-IPAQ) questionnaire (at the beginning and end) were interviewly completed. The lifestyle recommendations [34] which include 5% weight loss [45] and increasing moderate-intensity aerobic physical activity at least 3 times/week for 30–45 min [46] were advised at the beginning. The lifestyle changes which include being physically active and losing weight gradually are the common treatment of NAFLD [30, 31]. Therefore, the patients were advised to adopt these lifestyle changes to observe the medical ethics. These pieces of advice were equally presented to the all participants.

The values from 24-h food recall (valid in Iran [47]) were converted to gram per day [48]. The dietary status was determined using the *Nutritionist 4* software [47].

The levels of physical activity were low (< 600 MET-minutes/week), moderate (600 to < 1500 MET-minutes/week), and high (≥1500 MET-minutes/week) [49]. The SF-IPAQ questionnaire had been validated in Iran [50, 51].

#### Anthropometrics

Weight (at the beginning and end) and height (at the beginning) were assessed using a digital scale and stadiometer (*Seca® Germany, Model: 755 1,021,994*). Body mass index (BMI) was calculated by dividing the weight in kilograms by squared height in meters.

#### Sonography and serum factors

At the beginning and end of the study, the ultrasonography of liver was done after 12 h fasting state, by just one radiologist to reduce human error differences.

The 10 ml blood (at the beginning and end) collected from the peripheral vein after 12-h fasting during the night was centrifuged for 20 min (3000 *g*) and serum was frozen and stored at − 80 °C for analysis. The lab tasks (blood taking, storage, and tests) were done at the hospital.

Serum IL-6, TNF-α, and Sirt1 were determined using the ELISA kits by *Shanghai Crystal Day Biotech Co. Ltd®; Intra-assay CV < 8%, Inter-assay CV < 10*% and sandwich ELISA by an automatic device (*Elisys Uno Human®*) [52]. The sandwich ELISA by an automatic device (*Elisys Uno Human®*) for hs-CRP was done using ELISA kit by *Diagnostics Biochem Canada (DBC) Inc®, REF: CAN-CRP-4360, Version 5.0; Intra-assay CV ≤ 15.2%, Inter-assay CV ≤ 9.9%*. The serum levels of ALT and AST were measured using Hitachi analyzer device (*q17®*) and the specific kits as *Bionik®, Liquid Stable, NADH. Kinetic UV.IFCC, Intra-assay CV ≤ 4.27%, Inter-assay CV ≤ 4.68% and Bionik®, Liquid Stable, NADH. Kinetic UV.Liquid, Intra-assay CV ≤ 3.02%, Inter-assay CV ≤ 3.00%*, respectively.

### The sample size

The “two mean comparison formula” was used to calculate the sample size. In the previous study of the effects of cardamom on lipids, errors I and II, the mean difference of triglyceride (TG) between the groups, and the standard deviation of each group were 0.05 and 0.2, 5 mg/dl, and 8 mg/dl, respectively [44]. In all, 46 subjects were considered for both groups (GC and placebo) with a prediction of 15% sample drop.

### Data analysis and accessibility

The entry, security, coding, and storage of data were considered. The analysis was done by modified-intention to treat (m-ITT). The ITT population included all the randomized participants who received the allocated intervention. The missing data were imputed by using regression imputation method. The Kolmogorov-Smirnov test was used to determine normality of continuous variables. Chi-square, Fisher exact test, and t-test or Mann-Whitney test were used to assessed categorical and continuous baseline characteristics, respectively. Time effects and time × treatment interaction effects on all dependent variables were determined using two way repeated measures analysis of variance (TWRM-ANOVA). TWRM-ANOVA was adjusted for dietary intake of vitamin E and B6. The measurements were reported with 95% confidence interval (CI) and a *P*-value< 0.05 was considered as statistically significant. The Statistical Package for Social Sciences, version 16 (SPSS Inc., Chicago, IL, USA) and STATA_11SE_ (general-purpose statistical software package by StataCorp) software were used to analyze data. The access to the final dataset was possible only for the principal investigator and the results were given only by the publication.

## Results

### Characteristics of patients

According to the flowchart in Fig. 1, a total of 787 people were screened (medical history). One hundred and fourteen patients met the eligibility criteria, of whom 15 declined and 7 couldn’t participate. Ninety-two participants were randomized and 3 patients in GC group and 2 patients in the placebo group did not receive allocated intervention (refused to participate for personal reasons). Thus 87 participants completed the first visit (cardamom *n* = 43; placebo *n* = 44). Furthermore, 6 patients did not complete the study (for personal reasons and travelled; cardamom *n* = 3; placebo *n* = 3). In addition, the baseline values of blood biomarkers of one patient in the placebo group were not available. Finally, the data of 87 patients were analyzed according to the m-ITT.

The general characteristics, physical activity level, and the degree of fatty liver of 87 overweight or obese NAFLD patients are presented in Table 1. Patients had age and BMI of about 45 yrs. and 30 kg/m^2^, respectively and there were more numbers of male than female, married, employee or free job/retired, had nearly similar educations, in moderate and high economic levels, and low physical activity. The mean of supplement compliance by using capsule counting was more than 95% for both GC and placebo groups.

**Table 1:** General characteristics and physical activity level of overweight/obese patients with non-alcoholic fatty liver disease (NAFLD)

Variables			Cardamom (*n* = 43) n(%) or Mean(SD)	Placebo (*n* = 44) n(%) or Mean(SD)	*P*-value
Age (yrs)			45.5(8.9)	45.0(7.7)	0.7^*^
Gender	Male		27(62.8)	27(61.4)	0.8^**^
	Female		16(37.2)	17(38.6)	
Height (cm)			166.9(10.3)	169.5(9.5)	0.3^*^
Marriage status	single		4(9.3)	7(15.9)	0.3^**^
	married		39(90.7)	37(84.1)	
Job status	employee, free job/retired		32(74.4)	30(68.2)	0.5^**^
	housewife, unemployed		11(25.6)	14(31.8)	
Education level	up to associate degree		18(41.9)	20(45.5)	0.7^**^
	Bachelor and higher		25(58.1)	24(54.5)	
Economic level	Low (≤3 living items)		0(0)	0(0)	0.1^**^
	moderate (4–6 living items)		12(27.9)	6(13.6)	
	High (≥7 living items)		31(72.1)	38(86.4)	
Physical activity level (Baseline)	low (< 600 MET-minutes/week)		38(88.4)	34(77.3)	0.1^**^
	Moderate (600 to < 1500 MET-minutes/week)		5(11.6)	10(22.7)	
	High (≥ 1500 MET-minutes/week)		0(0)	0(0)	
Physical activity level (After 3 months)	low (< 600 MET-minutes/week)		29(67.4)	34(77.3)	0.2^$^
	Moderate (600 to < 1500 MET-minutes/week)		12(27.9)	6(13.6)	
	High (≥ 1500 MET-minutes/week)		2(4.7)	4(9.1)	
Fatty liver (Baseline)	No		0(0)	0(0)	0.1^**^
	Yes	Mild	27(62.8)	34(77.3)	
		Moderate	16(37.2)	10(22.7)	
		Severe	0(0)	0(0)	
Fatty liver (After 3 months)	No		18(41.8)	2(4.5)	< 0.001^**^
	Yes	Mild	22(51.2)	34(77.3)	
		Moderate	3(7.0)	8(18.2)	
		Severe	0(0)	0(0)	

*Mann-Whitney; **Chi-square; **$**Fisher exact test

Baseline characteristics were similar between patients, with the exception of the cardamom group that have higher dietary vitamin E intake (Tables 1, 2, 3).

**Table 2:** Comparison of baseline mean for weight, BMI, Sirt1, inflammatory factors, and liver enzymes in overweight/obese patients with non-alcoholic fatty liver disease (NAFLD)

Variables	Cardamom (*n* = 43) Mean(SD)	Placebo (*n* = 44) Mean(SD)	*P*-value
Weight (kg)	85.2(11.3)	88.6(13.2)	0.2^*^
BMI (kg/m2)	30.5(2.4)	30.7(3.2)	0.6^**^
Sirt1 (ng/ml)	1.3(0.5)	1.2(0.5)	0.6^*^
TNF-α (ng/l)	16.7(4.6)	15.9(3.4)	0.3^*^
IL-6 (ng/l)	9.7(3.0)	9.3(2.6)	0.5^*^
hs-CRP (mg/l)	5.5(3.0)	5.4(3.2)	0.8^**^
ALT (u/l)	44.5(16.4)	41.8(12.4)	0.4^**^
AST (u/l)	26.0(9.3)	25.1(8.0)	0.7^**^

*t-test; **Mann-Whitney; *BMI* body mass index, *Sirt1* sirtuin-1, *TNF-α* tumor necrosis factor-alpha, *IL-6* interleukin-6, *hs-CRP* high sensitive c-reactive protein, *ALT* alanine transaminase, *AST* aspartate transaminase

**Table 3:** Dietary intakes of overweight/obese patients with non-alcoholic fatty liver disease (NAFLD)

Dietary intakes		Cardamom (*n* = 43) Mean(SD)	Placebo (*n* = 44) Mean(SD)	*P*-value	
Energy (kcal)	Baseline	2309.3(763.3)	2164.6(761.6)	0.3^*^	0.4^**^
	1.5 Months	1857.8(563.7)	1871.1(641.1)	0.9	
	3 Months	1947.5(656.7)	1978.4(636.8)	0.8	
Protein (g)	Baseline	91.3(37.8)	83.7(34.4)	0.3	0.3
	1.5 Months	77.1(34.1)	74.8(31.6)	0.7	
	3 Months	79.6(31.7)	83.1(30.4)	0.7	
Protein (%)	Baseline	15.8(4.2)	15.5(4.1)	0.7	0.6
	1.5 Months	16.2(3.7)	15.9(3.7)	0.6	
	3 Months	16.4(4.4)	16.9(3.5)	0.5	
Carbohydrate (g)	Baseline	275.3(110.7)	261.1(94.0)	0.5	0.3
	1.5 Months	221.6(76.4)	234.6(80.6)	0.6	
	3 Months	231.1(90.3)	239.5(81.1)	0.6	
Carbohydrate (%)	Baseline	47.5(10.3)	48.7(9.8)	0.5	0.7
	1.5 Months	47.8(8.1)	50.5(6.6)	0.08	
	3 Months	47.3(7.8)	48.6(7.1)	0.4	
Fat (g)	Baseline	99.2(36.9)	89.9(43.9)	0.2	0.5
	1.5 Months	78.0(26.6)	75.1(30.6)	0.3	
	3 Months	82.3(29.6)	81.4(34.2)	0.5	
Fat (%)	Baseline	38.7(8.6)	37.2(8.4)	0.4	0.8
	1.5 Months	38.0(6.7)	35.8(5.2)	0.1	
	3 Months	38.2(6.3)	36.8(6.8)	0.3	
Cholesterol (mg)	Baseline	234.4(143.5)	221.9(174.5)	0.3	0.8
	1.5 Months	208.0(134.4)	186.1(141.6)	0.3	
	3 Months	290.6(375.1)	255.3(154.9)	0.4	
Saturated fat (g)	Baseline	26.0(11.2)	24.0(13.7)	0.4	0.7
	1.5 Months	21.2(9.6)	21.2(11.7)	0.7	
	3 Months	23.8(11.3)	22.8(8.7)	0.8	
Monounsaturated fatty acid (g)	Baseline	36.2(14.3)	32.9(16.9)	0.1	0.6
	1.5 Months	28.3(10.0)	27.5(11.4)	0.5	
	3 Months	30.5(11.2)	29.1(11.0)	0.3	
Polyunsaturated fatty acid (g)	Baseline	26.3(13.1)	22.4(12.2)	0.08	0.1
	1.5 Months	20.2(9.2)	19.5(8.8)	0.5	
	3 Months	19.2(8.6)	20.1(12.1)	0.7	
Vitamin A (RAE) (μg)	Baseline	465.7(449.5)	419.1(434.7)	0.4	0.3
	1.5 Months	285.1(222.8)	375.5(326.9)	0.2	
	3 Months	365.5(349.3)	397.2(246.2)	0.1	
Carotenoids (mg)	Baseline	10.3(9.3)	8.7(8.2)	0.3	0.2
	1.5 Months	6.9(6.7)	6.9(7.3)	0.8	
	3 Months	7.2(7.5)	8.9(6.3)	0.08	
Vitamin C (mg)	Baseline	88.7(92.1)	93.2(84.0)	0.7	0.7
	1.5 Months	84.6(83.4)	79.1(60.2)	0.8	
	3 Months	76.0(52.0)	87.1(61.9)	0.4	
Calcium (mg)	Baseline	968.3(589.4)	872.0(530.4)	0.3	0.05
	1.5 Months	925.4(390.3)	951.3(490.1)	0.7	
	3 Months	930.5(485.1)	1145.1(533.4)	0.03	
Iron (mg)	Baseline	15.1(6.6)	14.0(4.7)	0.4	0.7
	1.5 Months	11.6(4.0)	11.5(3.8)	0.8	
	3 Months	12.3(5.0)	11.7(4.4)	0.5	
Vitamin D (μg)	Baseline	0.9(1.4)	1.5(3.0)	0.7	0.7
	1.5 Months	0.9(1.5)	1.6(2.4)	0.1	
	3 Months	1.2(1.2)	2.1(2.1)	0.03	
Vitamin E (mg)	Baseline	31.7(16.2)	24.3(12.7)	0.01	0.01
	1.5 Months	23.8(11.2)	22.3(9.7)	0.4	
	3 Months	22.6(10.3)	23.8(11.3)	0.8	
Vitamin B1 (mg)	Baseline	1.6(0.7)	1.6(0.6)	0.6	0.7
	1.5 Months	1.4(0.5)	1.4(0.5)	0.9	
	3 Months	1.5(0.5)	1.5(0.5)	0.6	
Vitamin B2 (mg)	Baseline	1.9(1.0)	1.7(0.8)	0.6	0.06
	1.5 Months	1.6(0.6)	1.6(0.7)	0.9	
	3 Months	1.6(0.7)	2.0(0.8)	0.07	
Vitamin B3 (mg)	Baseline	27.0(13.5)	23.9(12.7)	0.2	0.4
	1.5 Months	21.8(12.9)	19.9(11.5)	0.4	
	3 Months	21.1(8.2)	21.9(10.3)	0.8	
Vitamin B6 (mg)	Baseline	1.9(0.9)	1.7(0.7)	0.2	0.02
	1.5 Months	1.6(0.7)	1.5(0.7)	0.4	
	3 Months	1.5(0.5)	1.7(0.6)	0.1	
Folate (DFE) (μg)	Baseline	405.7(192.2)	457.2(182.8)	0.8	0.5
	1.5 Months	370.8(176.5)	363.7(157.1)	0.6	
	3 Months	388.0(183.1)	348.8(130.9)	0.2	
Vitamin B12 (μg)	Baseline	4.1(2.5)	4.1(2.8)	0.8	0.9
	1.5 Months	3.5(2.1)	3.6(2.5)	0.9	
	3 Months	4.6(3.8)	4.4(2.0)	0.5	
Vitamin K (μg)	Baseline	257.3(489.8)	198.3(371.0)	0.5	0.4
	1.5 Months	118.9(225.8)	147.9(233.4)	0.2	
	3 Months	195.0(373.0)	113.2(193.2)	0.8	
Zinc (mg)	Baseline	11.8(5.2)	11.5(4.8)	0.9	0.7
	1.5 Months	9.7(3.6)	10.4(4.1)	0.5	
	3 Months	11.5(6.2)	11.4(3.8)	0.6	
Selenium (μg)	Baseline	98.9(48.1)	106.9(52.4)	0.4	0.7
	1.5 Months	85.6(42.3)	94.1(38.7)	0.3	
	3 Months	101.4(50.7)	103.3(43.5)	0.7	
Total fiber (g)	Baseline	30.3(16.5)	26.6(15.3)	0.2	0.3
	1.5 Months	26.1(13.1)	22.4(10.2)	0.1	
	3 Months	24.7(12.7)	25.2(12.2)	0.6	

*Total of the column: t-test or Mann-Whitney; **Total of the column: Two way repeated measures-ANOVA (TWRM-ANOVA)

### Changes in dietary intakes, degree of fatty liver, and blood biomarkers

The dietary intakes during the study were almost similar between groups, except for significantly higher dietary intake of vitamins E and B6 in the cardamom group (*P* < 0.05, Table 3) which were considered as confounders in the final analysis. Within cardamom group, the mean difference of AST was not significant (*P* > 0.05), whereas the weight, BMI, ALT, IL-6, TNF-α, and hs-CRP decreased and Sirt1 increased significantly (*P* < 0.05). Within placebo group, the mean difference of weight, BMI, hs-CRP, ALT, and AST were not significant (*P* > 0.05), but the IL-6 and TNF-α decreased and Sirt1 increased significantly (*P* < 0.05). At the end, compared to placebo, cardamom significantly improved fatty liver.

According to the time-by-treatment interaction effect in both unadjusted and adjusted analysis model, ALT, IL-6, TNF-α, and hs-CRP decreased and Sirt1 increased significantly among cardamom group in comparison with the placebo group (*P* < 0.05). The time-by-treatment interaction effect had trend for weight (*P* = 0.051) and BMI (*P* = 0.06) in the unadjusted model due to a small but non-significant decrease in the cardamom group more than the placebo group (Table 4).

**Table 4:** The changes of weight, BMI, Sirt1, inflammatory factors, and liver enzymes in overweight/obese NAFLD patients

Variables	Intervention	Baseline Mean(SD)	3 Months Mean(SD)	*P*-value^$^	Mean Difference (95% CI)	*P*-value^#^		
						Time	Treatment	Interaction
Weight (kg)	Cardamom (*n* = 43)	85.2(11.3)	84.2(11.3)	< 0.001	−1.0 (−1.7, −0.2)	< 0.001	0.1	0.051
	Placebo (*n* = 44)	88.6(13.2)	88.2(13.9)	0.2	−0.4 (−1.27, 0.47)	0.001	0.1	0.2
BMI (kg/m^2^)^*^	Cardamom (*n* = 43)	30.5(2.4)	30.1(2.4)	< 0.001	−0.4 (−0.55, −0.24)	< 0.001	0.8	0.06
	Placebo (*n* = 44)	30.7(3.2)	30.6(3.4)	0.1	−0.1 (− 0.31, 0.11)	< 0.001	0.8	0.2
Sirt1 (ng/ml)^**^	Cardamom (*n* = 43)	1.3(0.5)	1.7(0.5)	< 0.001	0.4 (0.36, 0.43)	< 0.001	0.054	< 0.001
	Placebo (*n* = 44)	1.2(0.5)	1.3(0.5)	0.003	0.1 (0.06, 0.13)	< 0.001	0.04	< 0.001
TNF-α (ng/l)	Cardamom (*n* = 43)	16.7(4.6)	8.7(4.3)	< 0.001	−8.0 (−8.2, −7.7)	< 0.001	0.001	< 0.001
	Placebo (*n* = 44)	15.9(3.4)	15.2(3.7)	0.03	−0.7 (−0.9, −0.4)	< 0.001	< 0.001	< 0.001
IL-6 (ng/l)	Cardamom (*n* = 43)	9.7(3.0)	5.0(2.6)	< 0.001	−4.7 (−4.8, −4.5)	< 0.001	0.008	< 0.001
	Placebo (*n* = 44)	9.3(2.6)	8.4(2.5)	0.001	−0.9 (−1.0, − 0.7)	< 0.001	0.009	< 0.001
hs-CRP (mg/l)^	Cardamom (*n* = 43)	5.5(3.0)	3.7(2.0)	< 0.001	−1.8 (− 1.9, − 1.6)	< 0.001	0.3	< 0.001
	Placebo (*n* = 44)	5.4(3.2)	5.3(3.0)	0.08	−0.1 (−0.3, 0.1)	0.01	0.4	< 0.001
ALT (u/l)^**^	Cardamom (*n* = 43)	44.5(16.4)	31.8(12.0)	< 0.001	−12.7 (−13.6, − 11.7)	< 0.001	0.1	< 0.001
	Placebo (*n* = 44)	41.8(12.4)	41.3(12.9)	0.5	−0.5 (−1.3, 0.3)	0.006	0.1	< 0.001
AST (u/l)^	Cardamom (*n* = 43)	26.0(9.3)	25.0(8.8)	0.08	−1.0 (−1.6, −0.4)	0.2	0.9	0.1
	Placebo (*n* = 44)	25.1(8.0)	25.2(7.6)	0.8	0.1 (−0.4, 0.6)	0.01	0.7	0.3

*Inversely transformed; **Transformed by square root; **^**Logarithmically transformed; **$**Paired t-test; **#**Two way repeated measures-ANOVA (TWRM-ANOVA), top row: unadjusted; bottom row: adjusted for vitamins E and B6 intake; *BMI* body mass index, *Sirt1* sirtuin-1, *TNF-α* tumor necrosis factor-alpha, *IL-6* interleukin-6, *hs-CRP* high sensitive c-reactive protein, *ALT* alanine transaminase, *AST* aspartate transaminase

### Safety

The patients reported no side effects associated with the treatment. Only one patient in the placebo group reported nausea and constipation in one of his follow up.

## Discussion

This trial for the first time assessed the effects of GC on blood inflammatory biomarkers, liver enzymes, and Sirt1 in overweight/obese NAFLD patients. According to time-by-treatment interaction effect in both unadjusted and adjusted analysis model, ALT, IL-6, TNF-α, and hs-CRP decreased and Sirt1 increased significantly among cardamom group in comparison with the placebo group. Moreover, the improvements in the degree of fatty liver in cardamom group was significantly higher than the placebo group. The decrease of the weight and BMI in cardamom group had a trend in comparison with the placebo group in unadjusted analysis model.

The results of different studies on the effects of cardamom or polyphenol-rich foods showed some controversies and some of them are presented as follows.

The polyphenols, especially resveratrol, are activators of Sirt1 [53, 54] and they can improve obesity [55]. In our study, cardamom increased the serum Sirt1 significantly; however, the decrease of weight and BMI were not significant. The reasons are likely short duration and lower sample size of the study. Furthermore, in an experimental study on streptozotocin-induced diabetic rats, treatment with low-, intermediate-, or high-polyphenol cocoa for 16 weeks (i.e., 0.12, 2.9 or 22.9 mg/kg/day of polyphenols) improved Sirt1 activity [56]. Sirts, particularly Sirt1, deacylate histones/proteins and have a number of activities including anti-inflammatory activity that may prevent and reduce the complications, development, and progression of NAFLD [19].

Serum level of Sirt1 was measured in this study. Sirt1 was primarily represented as a nuclear protein [57] and leukocytes (peripheral blood mononuclear cell [PBMC]) have been used for determining Sirt1 expression [58]. However, it was demonstrated that Sirt1 moves between the nucleus and cytoplasm. Sirt1 has been recently determined in the serum, in spite of the fact that its exact origin is unclear [57]. Serum Sirt1 has been reported as a potential biomarker for some diseases such as aging-associated diseases [57].

In two separate animal studies, cardamom significantly decreased weight gain and serum AST and ALT levels [59, 60]. In line with this study, the cardamom in diabetic patients for 8 weeks have no significant effects on measures of weight, BMI, waist, and hs-CRP [61]. This may be attributed to the type and form of the supplementation and the duration of the intervention.

Two cellular studies on cardamom demonstrated the suppression and inhibition of IL-6 and TNF-α release [62, 63]. The beneficial effects of cardamom on inflammatory factors including IL-6, TNF-α, and NF-κB [64–66] and serum AST and ALT levels [66–71] had been observed in most of the animal models.

Cardamom supplementation in hyperlipidemic, overweight, and obese pre-diabetic women for 8 weeks decreased serum hs-CRP level after adjustment of some covariates [72]. Another human study on the effects of daily consumption of Arabic coffee with two different doses of cardamom did not show significant alteration in the concentration of ALT, AST, and C-reactive protein (CRP). This could be attributed to the fact that participants were healthy subjects who had normal levels of CRP, and no indications of inflammation [73].

Cardamom’s bioactive principles (i.e. 1,8-cineol [eucalyptol], beta-pinene, geraniol) had been revealed to have anti-inflammatory activity by binding to TNF-α, IL-1 beta, IL-4, and IL-5 [74]. In addition, clinical trial reviews of natural products including ginger, turmeric, and other polyphenol-rich compounds [75, 76] and most studies on polyphenolic compounds have showed decreased levels of inflammatory factors including IL-6, TNF-α, and hs-CRP in healthy or non-healthy subjects [77–84].

The reasons why our results were in accordance or contrary to other studies can be attributed to the difference in the type and design of the study, the sample size, the type of disease, the type and form of supplementation, the intervention duration, and the higher or lower values of the serum profiles at the beginning of the study.

The reported mechanisms for beneficial effects of GC on inflammation include decreased infiltration of inflammatory cells, lipid peroxidation, and levels of advanced oxidation protein products (AOPP), increase of antioxidant enzymes activity [60, 67, 85], inhibition of inflammatory mediators including COX-2, iNOS, and NF-κB [40, 62, 63, 70, 86, 87, 88], decrease of hemolysis by the vitamin E deficiency [89], and increase of PPARγ activity and cytotoxicity of natural killer cells [74].

The explained mechanisms in the improvement of the liver enzymes and the degree of fatty liver by cardamom include decrease of lipid peroxidation, increase of antioxidant capacity, cardamom active contents including phenols, polyphenols, minerals (Cu, Mn) [59, 60, 67, 68, 89–91], 1,8-cineol [92], and flavonoids [70].

The observed effects of GC in NAFLD patients would make this study relevant. Although GC had been hypothetically used in the treatment of some disorders, it can be used in humans for further study in different diseases, particularly NAFLD. Also, the emerging rates of obesity and, consequently, NAFLD should be given more attention.

This study has several strengths. First, the double-blinded stratified blocked randomization design; Second, the inclusion of patients with newly diagnosed NAFLD who had not yet received treatment; Third, considering multiple eligibility criteria; Fourth, protocol publication, Fifth, the determining of dietary intakes and physical activity status and adjusting the statistical analysis for them and other potential confounders; Sixth, considering control group. These strengths are likely preferable in comparison with few other clinical trials that have evaluated the effects of any spices on NAFLD patients.

However, our study had some limitations. First, the sample size was small; Second, the intervention duration was short to understand the real effects of cardamom supplementation; Third, disregarding non-obese NAFLD patients. Fourth, self-reporting of diet and physical activity; Fifth, failure to perform liver biopsy and measure gamma-glutamyl transferase (GGT); Sixth, failure to check the bioavailability of GC and measure serum levels of its components; Seventh, 24-h food recall is not a good index for assessing the usual food intake; and Eighth, failure to measure body composition. Even so, this study is the first trial to evaluate the effects of GC on overweight/obese NAFLD patients.

## Conclusion

GC supplementation in obese NAFLD patients reduced inflammatory biomarkers (IL-6, TNF-α, and hs-CRP), ALT, and the degree of fatty liver and increased Sirt1 compared with placebo. Accordingly, GC may be useful in other metabolic diseases associated with inflammation. Further trials and considering the mentioned limitations are needed to confirm these results.

## Abbreviations

AOPP: Advanced oxidation protein products
BMI: Body mass index
CEO: Cardamomum essential oil
COX-2: Cyclooxygenase-2
ELISA: Enzyme-linked immunosorbent assay
GC: Green cardamom
GC-MS: Gas chromatography mass spectrometry
HPLC: High-performance liquid chromatography
hs-CRP: High-sensitivity C-reactive protein
IL-1β: Interleukin-1 beta
IL-6: Interleukin-6
iNOS: Inducible-nitric oxide synthase
IPAQ: International physical activity questionnaire
ITT: Intention to treat
NAFLD: Non-alcoholic fatty liver disease
NASH: Non-alcoholic steatohepatitis
NF-κB: Nuclear factor kappa-light-chain-enhancer of activated B cells
NIOC: National Iranian Oil Company
PGC-1α: PPAR-γ co-activator-1 alpha
PPAR: Peroxisome proliferation activated receptor
ROS: Reactive oxygen species
Sirt-1: Sirtuin-1
SOD: Superoxide dismutase
TG: Triglyceride
TMRC: Traditional Medicine Research Center
TNF-α: Tumor necrosis factor-alpha
TPN: Total parenteral nutrition
TWRM-ANOVA: Two way repeated measures-analysis of covariance
